# CK1 Is a Druggable Regulator of Microtubule Dynamics and Microtubule-Associated Processes

**DOI:** 10.3390/cancers14051345

**Published:** 2022-03-05

**Authors:** Aileen Roth, Adrian Gihring, Joachim Bischof, Leiling Pan, Franz Oswald, Uwe Knippschild

**Affiliations:** 1 University Medical Center Ulm, Department of General, and Visceral Surgery, University of Ulm, Albert-Einstein-Allee 23, 89081 Ulm, Germany; aileen.roth@uni-ulm.de (A.R.); adrian.gihring@uni-ulm.de (A.G.); joachim.bischof@uni-ulm.de (J.B.); 2 University Medical Center Ulm, Center for Internal Medicine, Department of Internal Medicine I, University of Ulm, Albert-Einstein-Allee 23, 89081 Ulm, Germany; leiling.pan@uni-ulm.de

**Keywords:** Casein Kinase 1, CK1, RITA, microtubule dynamics, cell cycle progression, microtubule transport, microtubule-associated proteins, MAPs, mitotic spindle, tumorigenesis

## Abstract

**Simple Summary:**

The Casein Kinase 1 (CK1) family of serine-threonine specific protein kinases regulates the activity of key regulatory proteins and signaling pathways being involved in embryonic development but also in the adult organism. Furthermore, it plays an important role in the regulation of proliferation, differentiation, apoptotic processes, circadian rhythm, chromosome segregation, and other microtubule-associated processes. Deregulation of CK1 expression and activity, as well as mutations in the coding region, contribute to the development of many human pathologies, including cancer. Alternations in the site-specific phosphorylation of α/β-tubulin and microtubule-associated proteins affect microtubule stability, finally resulting in mitotic defects and genomic instability. Here we review our knowledge about CK1 functions in general and especially in chromosome segregation. Furthermore, an update in modulating CK1 activity by small molecule inhibitors and peptides specifically inhibiting CK1 protein interactions as new therapy concepts for the treatment of cancer will be discussed.

**Abstract:**

Protein kinases of the Casein Kinase 1 family play a vital role in the regulation of numerous cellular processes. Apart from functions associated with regulation of proliferation, differentiation, or apoptosis, localization of several Casein Kinase 1 isoforms to the centrosome and microtubule asters also implicates regulatory functions in microtubule dynamic processes. Being localized to the spindle apparatus during mitosis Casein Kinase 1 directly modulates microtubule dynamics by phosphorylation of tubulin isoforms. Additionally, site-specific phosphorylation of microtubule-associated proteins can be related to the maintenance of genomic stability but also microtubule stabilization/destabilization, e.g., by hyper-phosphorylation of microtubule-associated protein 1A and RITA. Consequently, approaches interfering with Casein Kinase 1-mediated microtubule-specific functions might be exploited as therapeutic strategies for the treatment of cancer. Currently pursued strategies include the development of Casein Kinase 1 isoform-specific small molecule inhibitors and therapeutically useful peptides specifically inhibiting kinase-substrate interactions.

## 1. Introduction

Microtubules are helical fibers with an outer diameter of approximately 25 nm that consist of tubulin subunits (heterodimer of α- and β-tubulin) [1]. They present one of the main components of the eukaryotic cytoskeleton responsible for cell stability but also play a critical role in cell motility, intracellular transport, and mitosis [2]. Microtubules are organized by microtubule-organizing centers (MTOCs), including centrosomes, which serve as important nucleating factors that initiate microtubule polymerization [3,4]. The centrosome consists of two centrioles, surrounded by the pericentriolar material (PCM), which contains components such as γ-tubulin, which is important for anchoring and nucleating cytoplasmic microtubules to build up the mitotic spindle during cell division [5]. The mitotic spindle is organized by more than 1000 microtubule-associated proteins (MAPs), indispensable for the controlled regulation of the mitotic spindle [6].

If mitotic processes are not proceeding correctly, unequal distribution of chromosomes to the daughter cells and aneuploidy could have severe consequences, which might exert tumor-promoting functions. Therefore, mitosis needs to be strictly regulated and controlled. These regulatory processes are mainly driven by components of the mitotic kinome, including several kinase families such as NIMA-related kinases (Neks), cyclin-dependent kinases (CDKs), Polo-like kinases (Plks), and Aurora kinases, as well as phosphatases and kinase inhibitors [7]. More than 1000 phosphoproteins have been detected to be regulated in a cell cycle-dependent manner, and site-specific phosphorylation (in-) activates mitotic proteins or might even target them for degradation [8,9].

Major kinases regulating the microtubule network belong to the Aurora and Plk families. The primary function of Aurora kinases is the control of cell division. Two pools of Aurora A (AurA) are involved in the regulation of mitosis: a first one supporting centrosome maturation in the G2 stage of the cell cycle and a second one supporting assembly and proper function of the bipolar spindle by associating with centrosome-proximal microtubules in metaphase [10]. Aurora B (AurB) and Aurora C (AurC) are involved in chromosome condensation, kinetochore attachment, and alignment of chromosomes in later stages of mitosis [11]. In several aneuploid human tumors (including breast, colorectal, hepatic, lung, and oral cancer), amplification, overexpression, or hyperactivation of AurA and AurB can be found; however, high levels of AurB might rather be a consequence than the cause of malignant transformation [12,13,14]. Similar to Aurora kinases, Plks are also involved in the regulation of cell division by controlling essential mitotic processes. Plk1 and Plk4 are the most studied Plks, and while Plk1 regulates centrosome maturation, spindle formation, and cytokinesis, Plk4 is important for controlling centriole division [15,16]. Apart from the regulation of centrosome- and spindle-associated processes, Plk1 is also able to phosphorylate p53, thereby initiating its degradation [17].

Besides Aurora kinases and Plks, CDKs and Neks also play critical roles in cell cycle regulation by phosphorylating multiple mitosis-related substrates. CDKs are known to be activated at each stage of the cell cycle by the formation of stage-specific cyclin/CDK complexes [18]. After activation, cyclin/CDK complexes promote DNA replication, centrosome duplication, spindle formation, and other cell cycle-associated processes by the phosphorylation of mitotic key regulators [19]. Members of the Nek family are initially characterized by their function in the regulation of mitosis, primarily including DNA damage response, cell cycle regulation, and centrosome organization [20,21,22,23,24,25].

In addition to the abovementioned kinase families, members of the CK1 family of protein kinases are also known to be involved in the regulation of mitotic processes. The CK1 family is evolutionary highly conserved, ubiquitously expressed, and constitutes one of the first serine/threonine-specific protein kinases discovered [26,27]. The CK1 isoforms α, β, γ1, γ2, γ3, δ, ε, together with the closely related Tau Tubulin Kinase 1 (TTBK1) and the Vaccine-related kinases (VRKs), form their own independent branch of the kinome tree of eukaryotic protein kinases [28,29]. Due to their wide range of substrates, CK1 isoforms are involved in many developmental pathways, including Wnt (Wingless/Int-1), Hh (Hedgehog), and Hippo signaling pathways, which are important in growth, homeostasis, and tissue development. Mutations of components and aberrant regulation of these pathways have been connected to various cancer entities [26,30]. The following section concentrates on the current knowledge of the contribution of CK1 in tumorigenesis and tumor progression.

## 2. Participation of CK1 in Tumorigenesis and Tumor Progression

**Figure 1:**
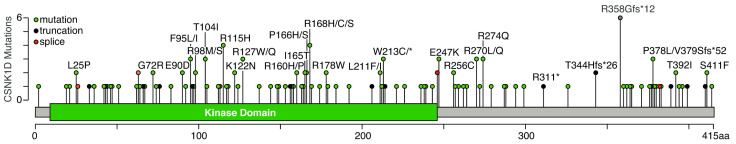
Mutations in CSNK1D (CK1δ). According to cBioPortal for Cancer Genomics, 852 different mutations have been reported in a curated set of 202 nonredundant studies, including 90,279 samples [31,32]. Positions of mutations in the CK1δ protein are shown. Highlighted mutations (*n* > 1) and tumor samples are summarized in Table 1. Amino acids are shown in one-letter code. Abbreviations: aa—amino acids, *—stop codon, fs—frame shift.

Cancer-related functions of the CK1 family are closely connected to the role of CK1 in the abovementioned signaling pathways. Besides that, numerous studies substantiated the oncogenic potential of CK1 by findings that CK1 isoforms modulate key regulatory proteins such as β-catenin, MDM2, and p53, which can be seen as crucial regulators in tumorigenesis [26,27]. So far, various mutations within CSNK1D encoding for CK1δ have been identified and observed in different types of cancer. According to cBioPortal for Cancer Genomics, 852 different mutations have been reported in a curated set of 202 nonredundant studies (including 90,279 samples) (Figure 1 and Table 1).

**Figure 2:**
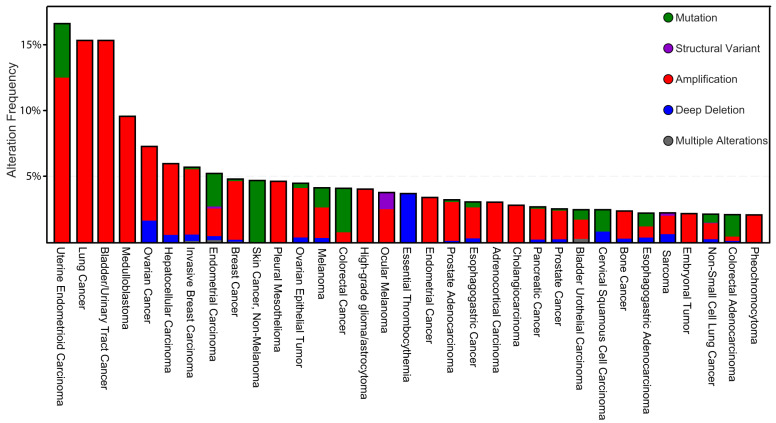
Genomic alternation frequencies and tumor types affecting the CSNK1D gene. 33 different tumor entities (Alternation Frequency ≥ 1%) were analyzed using the cBioPortal for Cancer Genomics [31,32] accessing the actual TCGA dataset. The highest genomic amplification frequency for CSNK1D was detected in Lung Cancer and Bladder/Urinary Tract Cancer (approx. 15%).

Although the mutation rate of CSNK1D is very low, a TCGA database analysis from certain tumor tissues and tumor cell lines clearly indicates genomic amplification of CSNK1D with the highest frequency in lung cancer and bladder/urinary tract cancer (Figure 2). Genomic alterations could explain that alterations in the expression levels in different cancer entities including urinary tract/bladder cancer [33], lung cancer [34], colorectal cancer [35], breast carcinomas [36], ductal pancreatic carcinomas [37], and blood cancer [38,39] (reviewed in [40,41]) contribute to tumorigenesis and tumor progression.

Several studies provide evidence that the CK1 family exhibits oncogenic potential by promoting genome instability, promoting proliferation, and inhibiting apoptotic processes (reviewed in [42]), which is provoked by increased kinase activity caused by mutations in CSNK1D and, in particular, by overexpression of CK1δ in tumors.

Within the last few years, several CK1 isoforms were shown to be involved in the regulation of mitotic spindle organization and mitotic processes. Since cancer is characterized by uncontrolled cellular proliferation, which is caused by the aberrant activity of various cell cycle regulating proteins, cell cycle regulators, such as CK1, are seen as interesting targets in cancer therapy. A detailed presentation of CK1 isoforms in regulating cell cycle progression, modulating cytoskeleton components, and the role in microtubule transport will be described in detail in the following chapters.

## 3. The Role of CK1 in Cell Cycle Progression

Members of the CK1 family are known to be important regulators of genomic stability, microtubule dynamics, cell cycle progression, mitosis, and meiosis [43,44,45,46,47,48,49,50,51,52,53,54]. Interestingly, the *Saccharomyces cerevisiae* orthologue of CK1δ, Hrr25, was one of the first kinases being described to regulate cell cycle progression [55]. Recently, a study demonstrated that the inhibition of Hrr25 led to the assembly of unusually long cytoplasmic microtubules and incorrect spindle positioning [56]. P-bodies, cytoplasmic RNA-proteins (RNP), were found to provide protection for Hrr25 and CK1 in meiotic cells. Inhibition of this interaction led to decreased levels of Hrr25 and disturbed meiosis progression [57,58]. In addition to the *S. cerevisiae* orthologue Hrr25, the *Schizosaccharomyces pombe* orthologues of CK1, Hhp1, and Hhp2, have also been reported to be involved in the mitotic checkpoint by delaying cytokinesis under mitotic stress. In the context of these studies, it was shown that CK1 localizes to the spindle pole bodies (SPBs) and thereby phosphorylates septation initiation protein 4 (Sid4), leading to its degradation and cytokinesis suspension [47,59].

**Figure 3:**
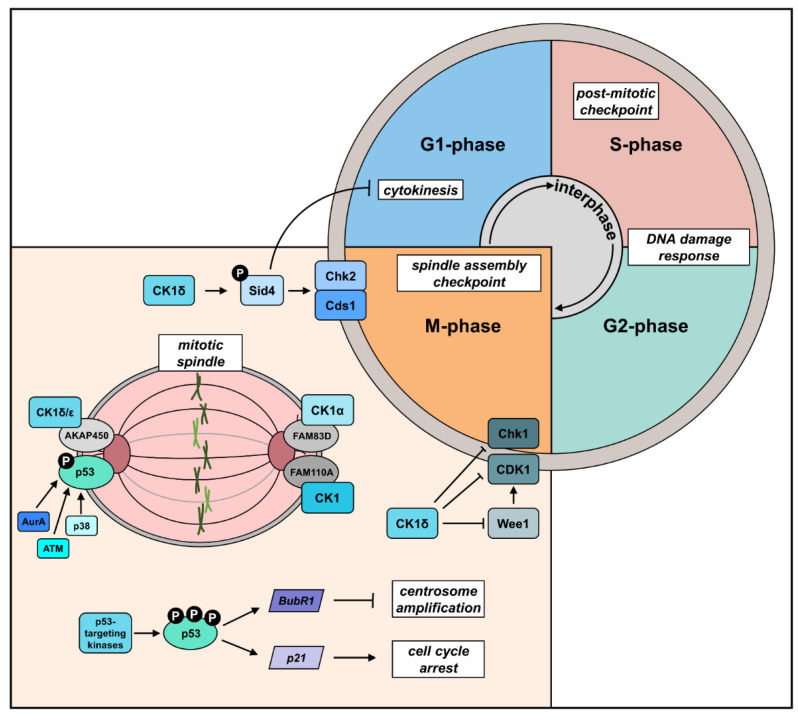
Cell cycle-associated functions of the CK1 family. CK1 localizes to spindle pole bodies and thereby phosphorylates Sid4 leading to its degeneration and delayed cytokinesis. Additionally, phosphorylation of Sid4 causes the recruitment of Chk2/replication checkpoint kinase Cds1, which supports the mitotic commitment. CK1α, CK1δ, and CK1ε are localized to the mitotic spindle mediated through the interaction with centrosome-associated proteins, such as AKAP450, FAM83D, and FAM110A. Site-specific phosphorylation of p53 leads to p53-dependent transcriptional activation of target genes such as p21 and BubR1 involved in the regulation of centrosomal functions, such as centrosome amplification and cell cycle arrest. Silencing of CK1δ leads to decreased expression of CDK1 and Chk1, which are both involved in mitotic checkpoints and DNA damage response. Moreover, CK1δ-mediated degradation of Wee1 increases levels of active CDK1 and, thus, initiate the entrance of cells into mitosis. Parts of the figure were generated using templates from Servier Medical Art [69], which is licensed under a Creative Commons Attribution 3.0 Unported License.

Unfortunately, the precise contribution of each mammalian CK1 isoform to central functions in the regulation of the cell division cycle is not well understood. However, CK1δ was shown to be associated with the centrosome, kinetochore, and microtubules, pointing to a cell cycle checkpoint control function of CK1δ [43,46]. The hypothesis that CK1 fulfills regulatory roles at the centrosome is further supported by the findings that CK1δ and CK1ε interact with the scaffold protein A-kinase anchor protein 450 (AKAP450). AKAP450 acts as an anchor point for CK1δ at the centrosome enabling the CK1-mediated phosphorylation of the microtubule plus-end-binding protein 1 (EB1) and presenting a relevant factor for centrosome positioning during T cell activation [60] (Figure 3). Moreover, silencing of CK1δ led to decreased expression of the cell division cycle 2 (CDC2)/CDK1 and checkpoint kinase (Chk)1, both being involved in mitotic checkpoints and DNA damage response [54]. Interaction of CK1δ with Chk1 and Chk1-mediated regulation of CK1δ activity have previously been shown [61]. Additionally, CK1δ-mediated degradation of Wee1-GC checkpoint kinase (Wee1) induced increased levels of active CDK1 and, thus, the entrance of cells into mitosis [50] (Figure 3). Interestingly, inhibition or depletion of CK1δ provoked reduced Wee1 turnover, increased phosphorylation of CDK1, and, as a consequence, cell cycle exit [49,50]. However, it has still to be proven if the centrosome-associated fraction of CK1δ is involved in mediating this control. This assumption is supported by the fact that CK1δ-mediated phosphorylation of phosphoprimed Sid4, a scaffold protein and anchoring point of the spindle pole body in fission yeast, triggers the recruitment of Chk2/replication checkpoint kinase Cds1 [53] (Figure 3).

A far greater role with regard to spindle positioning and mitosis is played by CK1α. An immunohistological approach showed that CK1α localizes to mitotic spindles [62], and injection of CK1α-specific morpholinos caused mitotic arrest and chromosomal misalignments in mouse oocytes [63]. A role in spindle positioning and cell division was shown for the interaction of CK1α with the FAMily of sequence similarity (FAM)83 [64]. A recent study demonstrated that CK1α is recruited to the mitotic spindle by FAM83D, and CK1α-binding deficient FAM83D^F283A/F283A^ knocking mutations exhibit prolonged mitosis and spindle positioning defects [65]. However, stronger interaction with CK1α was observed for FAM83B, FAM83E, FAM83G, and FAM83H detected via immunoprecipitation and mass spectrometry [66]. Recently, a study assumed that mutations in FAM83G, more precisely in the conserved domain of unknown function 1669 (DUF1669), lead to disruption of CK1α interaction and, thereby, attenuation of Wnt signaling [67]. Similar results have recently been shown for FAM110A. In this study, it was demonstrated that CK1 interacts with FAM110A during mitosis, and inhibition of CK1 or depletion of FAM110A, led to chromosomal alignment defects and delayed mitosis progression (Figure 3). Interestingly, defects in chromosomal alignment were rescued by mimicking phosphorylation with FAM110A-S252-255E mutants [68]. Functional binding partners of CK1, such as the anchoring proteins FAM83 and FAM110A, could be used as alternative targets in cancer treatment by blocking specifically the interaction of CK1 isoforms with these anchoring proteins and thus, inhibiting CK1 isoform-mediated substrate phosphorylation (see also Section 7.4) [68].

## 4. The Contribution of CK1 to the Modulation of Cytoskeleton Components

**Figure 4:**
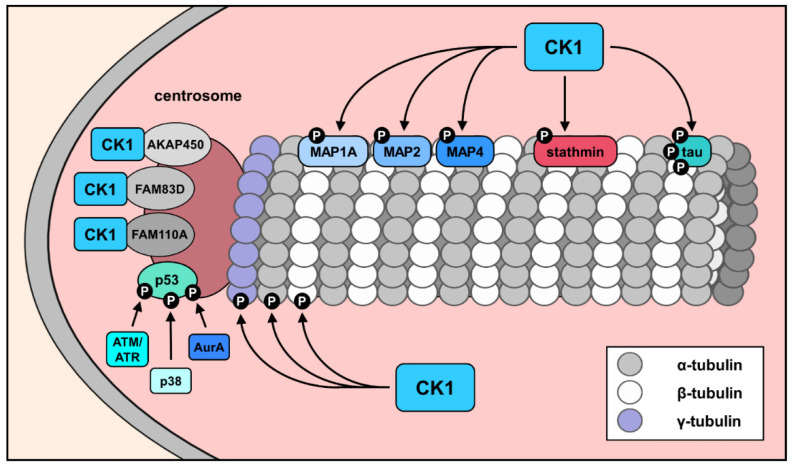
The role of CK1 in the modulation of cytoskeleton components. Direct CK1-mediated phosphorylation of microtubule subunits, such as α-, β-, and γ-tubulin, leads to the modulation of microtubule polymerization, stability, and spindle dynamics. In addition, microtubule dynamics are also influenced by CK1-mediated phosphorylation of MAPs, such as MAP1A, MAP2, MAP4, stathmin, and tau [44,74,75,76] (Figure 4). Parts of the figure were generated using templates from Servier Medical Art [69], which is licensed under a Creative Commons Attribution 3.0 Unported License.

Due to their numerous substrates, it is not surprising that CK1 isoforms have also been implicated in the modulation of microtubule polymerization, stability, and spindle dynamics linked to the direct phosphorylation of the microtubule subunits α-, β-, and γ-tubulin [44,46]. In synchronized mitotic cells treated with DNA damaging agents (such as etoposide or camptothecin), the association of CK1δ with α- and γ-tubulin increases [44], indicating its regulatory functions at the mitotic spindle. A function for CK1 in spindle association, microtubule modulation, and microtubule dynamics by phosphorylation of several MAPs, which have prognostic relevance for the overall survival of cancer patients, was also shown [65,70,71,72,73]. So far, several MAPs, such as MAP1A, MAP2, MAP4, stathmin and tau were discovered to be phosphorylated by CK1 [44,74,75,76] (Figure 4). The most studied CK1 interaction partner within the MAPs is the tau protein. Recent studies demonstrated the importance of CK1 isoforms in the abnormal hyperphosphorylation and deregulation of tau, finally leading to microtubule destabilization and neuronal cell death, which is associated with Alzheimer’s disease [77]. Moreover, the involvement of tau in the regulation of cell migration has been reported by more recent studies and reviewed in [78]. Increased levels of tau phosphorylation in cancer cells, subsequent detachment of tau from microtubules, and its inability to perform microtubule-related functions have been described in several studies. Hyperphosphorylated tau has been detected in prostate and colon cancer cell lines. Additionally, increased levels of phospho-tau were shown to be a marker for nonmetastatic colon cancer [79,80,81].

The involvement of CK1 in microtubule dynamics is also supported by the close connection of CK1δ to microtubule-associated centrosomal subfraction of the tumor suppressor protein p53 [82,83,84] (Figure 4). Generally, p53 is involved in centrosome duplication and, therefore, protective against defective centrosome amplification and reduplication, preventing the occurrence of mitotic errors and the development of chromosomal instability [84,85,86,87,88]. In order to meet these demands, p53-dependent transcriptional activation of target genes, such as p21 and BubR1 (regulating centrosome functions) [89,90], as well as transcription-independent functions of p53, are required. The latter includes the ability of p53 to interact with several centrosome-associated proteins, such as γ-tubulin and centrin. These interactions appear to be necessary to maintain centrosome biogenesis [91]. Site-specific phosphorylation of p53 seems to play an important role in this function. Thus, phosphorylation of p53 at Ser15 is essential for colocalization with various centrosomal proteins, thereby contributing to the inhibition of uncontrolled centrosome duplication [92]. There is evidence that site-specific phosphorylation of p53 at Ser15, and Ser33 by centrosome-associated and stress-induced kinases, such as AurA, ATM/ATR, and p38, is required for the maintenance of centrosome homeostasis under normal and various stress conditions [87,93,94,95,96]. Furthermore, the ability of p53 to bind to unduplicated centrosomes depends on the phosphorylation of Ser315 by CDK2 and might also be important for p53-mediated regulation of centrosome duplication [84].

In addition, since both p53 and CK1 isoforms interact with the centrosome, new insights are needed concerning the role of CK1δ/ε-mediated site-specific phosphorylation of p53 in modulating the centrosomal functions of p53, such as the association of p53 with key centrosome factors necessary for inhibition of the duplication of centrioles [97]. A particular focus should also be placed on the implications of the functional significance of the interactions of CK1δ with centrosomal p53, as there is evidence that CK1δ and p53 are linked by an autoregulatory feedback loop [97].

## 5. CK1-Associated Functions in Microtubule Transport

CK1 plays a role in regulating cell cycle progression and the interactions between microtubules and membranes. In addition to that, several studies proposed that CK1 isoforms affect transport processes along microtubules. Recently, it was demonstrated that CK1ε is involved in the regulation of dynein-dependent transport processes by phosphorylation of the dynein intermediate chain component IC138, thereby inhibiting the minus-end directed transport of membrane organelles [98,99]. Interestingly, CK1 inhibitors rescued dynein activity, which was blocked by the phosphorylation of IC138. The hypothesis that CK1 has an inhibitory role in microtubule transport was also supported by the findings that solubilization of CK1 and the use of CK1-specific inhibitors restored microtubule sliding in *pf17* (paralyzed flagellar mutant) axonemes [100].

## 6. The MAP RITA as a Putative Target for CK1

RITA (RBP interacting and tubulin-associated), a highly conserved 36 kDa protein, was originally identified in a yeast to hybrid screen searching for novel RBPJ interacting proteins [101]. The transcription factor RBPJ also called RBP or CSL, is the central DNA-binding hub of the highly conserved Notch signaling pathway [102]. Notch signaling regulates fundamental cellular processes during embryonic development and in the adult organism. Aberrant Notch signaling results in severe congenital diseases and cancer [103,104]. After ligand binding to the Notch receptor, signal transduction involves the presenilin-dependent intracellular processing of the receptor and nuclear translocation of its intracellular domain (NICD). NICD associates with RBPJ and recruits coactivator complexes to activate transcription. In the absence of a Notch signal, RBPJ recruits corepressor complexes to shut down transcription [105].

**Figure 5:**
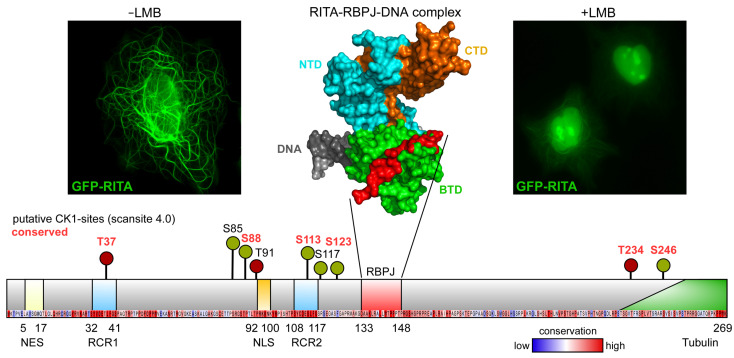
RITA is a tubulin- and RBPJ-binding shuttle protein with putative CK1δ phosphorylation sites. GFP-RITA shows association to tubulin fibers (-LMB, **left**) and localizes to the nucleus after inhibition of nuclear export by leptomycin B (+LMB) (**right**). Structure of the DNA bound RBPJ-RITA complex (**middle**), (PDB-ID: 5EG6). RITA (red) interacts with the beta-trefoil domain (BTD, green) of RBPJ in a RAM-like association. Scansite 4.0 identifies several putative CK1δ phosphorylation sites. Conserved sites in several species are marked in red. Human RITA is a 36 kDa protein with 269 amino acid residues. Identified domains are specified. NES, nuclear export signal, RCR1 and 2, RITA conserved repeat 1 and 2, NLS, nuclear localization signal, RBPJ, RBPJ interaction domain, Tubulin, tubulin-binding domain. Conserved amino acid residues are shown under the schematic protein representation.

RITA binds to the beta trefoil domain (BTD) of RBPJ, and in a recent structure–function analysis, we identified this type of interaction as a “RAM-Type” (Figure 5, upper middle panel) since it shows a striking similarity to the interaction of RBPJ with the RAM domain (RBPJ-associated molecule) of NICD [106] and now explains the mutually exclusive binding of either RITA or NICD to RBPJ on the structural level [107].

Further analysis revealed RITA not only interacts with RBPJ but also binds to tubulin in the cytoplasm and shuttles rapidly between the cytoplasm and the nucleus [101]. (Figure 5, upper left and right panel). We could identify and functionally characterize a nuclear export signal (NES) as well as a nuclear localization signal (NLS) and the tubulin-binding domain within the RITA proteins of various species (Figure 5, lower). Mechanistically, RITA interferes with RBPJ-NICD interaction and exports RBPJ from the nucleus to down-regulate Notch activity.

Interestingly, RITA-deficient mice show no obvious developmental defects, but aged animals develop tissue infiltrating lymphomas. In addition, RITA down-regulation was also found in hepatocellular carcinoma (HCC), suggesting that RITA might be a novel tumor suppressor [108,109].

In a recent study, we found that RITA localizes to interphase microtubules as well as to mitotic microtubule structures, thereby “bundles” the microtubules “thickened” fibers as shown by high-resolution microscopy. Cells deficient of RITA show altered microtubule stability together with highly acetylated α-tubulin. Microtubule dynamics is reduced in cells after RITA knockdown as well as in RITA-deficient mouse embryonic fibroblasts (MEF), leading to mitotic defects such as chromosome segregation errors and chromosome misalignment.

From this study, it is suggested that RITA recruits histone deacetylase (HDAC)-6 to tubulin, and after knockdown of RITA, increased activity of microtubule-associated acetyltransferase Mec-17 leads to an increase of tubulin acetylation and stabilization [110]. Furthermore, RITA was shown to be involved in the activation of AurA kinase activity at spindle poles [111] and in the regulation of cellular migration and invasion [112]. Thus, RITA might play a novel critical role in modulating microtubule dynamics, and its deregulation may contribute to chromosome instability and tumorigenesis.

To get a deeper insight into the regulation of RITA by posttranslational modifications, especially by phosphorylation, putative kinase target sites have been identified by Scansite 4.0 analysis [113]. Interestingly, a database search revealed various putative CK1 phosphorylation sites (Figure 5). Additional analyses are necessary to identify the exact phosphorylation sites of CK1δ on RITA and the functional consequences. However, RITA might be an additional target of CK1δ as shown for the MAPs MAP1A, MAP2, and MAP4 [44,74], and CK1δ-induced deregulation of RITA might also have an impact on tumor initiation and progression.

## 7. Addressing Inhibition of Cell Division in Cancer Therapy

### 7.1. Microtubule-Targeting Agents

Since the first approval of vinca alkaloids and taxanes for therapeutic application in the 1960s, the microtubule skeleton has emerged as an important target for anticancer therapy. Generally, two main categories of microtubule-targeting agents (MTAs) (also known as tubulin-binding agents, TBAs) can be distinguished: microtubule-stabilizing agents (MSAs), such as taxanes, increase the lateral interactions between the tubulin heterodimers, consequently resulting in increased polymerization and stabilization of microtubules. Microtubule-destabilizing agents (MDAs), such as colchicines and vinca alkaloids, lead to microtubule depolymerization by decreasing or inhibiting (mainly, but not only) longitudinal interactions between tubulin heterodimers. These MTAs interact with tubulin via six different binding sites. While the taxane, laulimalide/peloruside, vinca, and maytansine sites are located on β-tubulin, the colchicine site is located in proximity to the interface between the α- and β-subunits, and the pironetin site is located on the α-tubulin subunit (reviewed in [114]). So far, various MTAs have been already approved or are currently in clinical investigation phases for cancer treatment (reviewed in [115]). 

Most MTAs have been isolated from plant, fungi, or invertebrate origin with paclitaxel (isolated from *Taxus brevifolia*) [116], vinca alkaloids (discovered in *Catharantus roseus* (L.) G. Don) [117], and colchicine (isolated from autumn crocus *Colchicum autumnale*) [118] being the best-known MTA compounds. While the use of paclitaxel and vinca alkaloids in anticancer therapy is associated with severe side effects such as neurotoxicity, myelosuppression, or the development of multidrug resistance, the discovery of less toxic derivatives of these compounds enabled successful treatment of various cancers including breast, lung, bladder, prostate, and other cancers (reviewed in [119]). Due to its severe toxicity, neither colchicine nor any other colchicine-site MTA has been approved for cancer treatment so far, although some promising derivatives are currently under investigation in clinical trials [120]. New MTAs of natural or synthetic origin are still to be discovered and tested in (pre-) clinical investigation. Combretastatin and its analogs (such as Ombrabulin, first isolated from *Combretum caffeum*) demonstrated potent antitumor activity and safety in the treatment of ovarian cancer [121,122,123,124]. Epothilones belong to the microtubule-stabilizing group of MTAs, being first discovered as antifungal agents produced by *Sorangium cellulolus* ([125] and references therein). The mechanism of epothilones is similar to paclitaxel, and the epothilone derivative Ixabepilone has already been approved for the treatment of aggressive metastatic or locally advanced breast cancer [126,127]. The synthetic sulphonamide ABT-751 binds to the colchicine binding site on β-tubulin and inhibits microtubule polymerization. Significant anticancer effects, which have been demonstrated against non-small cell lung cancer and colon cancer, could be obtained by blocked cell cycle progression and induced apoptosis [128,129].

However, MTAs still present certain drawbacks, such as poor solubility, low bioavailability, toxicity, and multidrug resistance. Neurological side effects, including peripheral, cranial, and autonomic neuropathy as well as headache, dizziness, and mental depression, and hematologic side effects referred to as myelosuppression are the main toxicities associated with MTAs and often have dose-limiting consequences. Common side effects also include nausea, vomiting, and diarrhea [130]. Approaches to limit adverse events but also to overcome resistance, therefore, include combination therapy, e.g., paclitaxel and gemcitabine for the treatment of advanced pancreatic cancer [131] and numerous other combinations being currently under clinical investigation (reviewed by Nawara et al., [132]), as well as introducing novel antibody drug conjugates such as ado-trastuzumab emtansine (the maytansine derivative emtansine was conjugated to trastuzumab), which has recently been evaluated and approved for anticancer therapy [133]. As an alternative to directly targeting microtubules, an intervention on microtubule dynamics can also be achieved by targeting the mitotic kinome responsible for posttranslational modifications of microtubules or MAPs.

### 7.2. Inhibitors Targeting the Mitotic Kinome

The ATP-competitive AurA-selective inhibitor Alisertib (MLN8237) induces cell cycle arrest in the G2/M phase, apoptosis, and autophagy [134,135,136,137] (see Table 2). Alisertib prevents AurA-induced stabilization of N-Myc [138] and is currently under clinical investigation for the treatment of various malignancies, including neuroblastoma, small cell lung cancer, neurocrine prostate cancer, and breast cancer, among others [139,140,141]. The AurB-selective inhibitor Barasertib (AZD1152) inhibits tumor growth by decreasing histone phosphorylation resulting in the accumulation of aneuploidy cells and induction of apoptosis [142,143]. Cytotoxic effects of Barasertib might also be associated with stimulation of reactive oxygen species (ROS) production [144]. Barasertib has been tested in clinical trials for acute myeloid leukemia (AML) but induced severe side effects. Improved efficacy and tolerability are now expected for a new Barasertib nanoparticle formulation [145]. Apart from these compounds, a non-ATP-competitive inhibitor of AurA has also been described (AurkinA) binding to the Tpx2-binding surface of AurA and consequently displacing AurA from the mitotic spindle [146].

Volasertib, the most studied ATP-competitive inhibitor of Plk1, arrests cells in the G2/M phase and subsequently induces apoptosis [147,148]. Clinical trials demonstrated that Volasertib is more potently inhibiting the growth of hematopoietic malignancies in comparison with solid tumors [149].

Similar to Aurora kinases and Plks—and as introduced in the previous sections—members of the CK1 family are also involved in regulating microtubule dynamics and mitotic processes via their interaction with centrosomes, the phosphorylation of microtubule-associated cellular components, and their recruitment to the mitotic spindle apparatus. Therefore, also CK1 appears to be an attractive drug target for the induction of anti-cancer effects mediated by interference with microtubule-related processes. Since the association of CK1δ is significantly enhanced by treatment of the cells with DNA damage induced by camptothecin, etoposide, or γ-irradiation [44], simultaneous treatment with CK1δ-specific inhibitors might have synergistic or additive effects. Pharmacological inhibition, as well as siRNA-induced knockdown of CK1δ, already demonstrated effective inhibition of primary ciliogenesis via negative regulation of centrosome-specific functions and inhibition of (AKAP450-dependent) microtubule nucleation at the Golgi apparatus [48]. However, the specific functions mediated by distinct CK1 isoforms and the effects of CK1 isoform-specific inhibition need to be investigated carefully in order to obtain the desired anticancer effects.

Furthermore, therapeutic effects could also be achieved by intervening with CK1-mediated phosphorylation of MAPs such as Tau. Tau might be able to influence tumorigenesis by abnormal modulation of cell cycle progression, cell mobility, or organelle organization, and in fact, neurons from patients suffering from neurodegenerative diseases, including characteristic tau pathology, display hallmarks of DNA replication and active cell cycle as well as microtubule-mediated deformation of the nucleus [79,80,81]. Consequently, treatment with CK1-specific inhibitors (described in Table 2) could have therapeutic potential in cases where hyperphosphorylated tau can be linked to tumorigenesis, and reduction of Tau phosphorylation level has already been achieved by treatment of cells with CK1-specific inhibitors or CK1-specific siRNA [77,150]. In addition, more recently published potent CK1 isoform-selective small molecule inhibitors (SMIs) could prove to be therapeutically active (for review, see [41,42,151]). However, since treatment with CK1-specific inhibitors might improve tau binding to tubulin, these inhibitors may not be used together with taxanes because tau has been shown to interfere with the binding mode of taxanes to tubulin [152,153].

A special focus is given to the inhibitor IC261, which is one of the first potent SMI for CK1 first published in 2000, demonstrating obvious anticancer effects in subsequent studies within the following ten years [37,46,154,155]. However, it was observed that mitotic arrest in prometaphase and cytotoxicity is induced by CK1δ- and ε-independent effects of IC261. In comparison with different compounds inducing prometaphase arrest, the effects induced by IC261 were similar to those observed for nocodazole and colchicine. Moreover, IC261 even competed with colchicine for its binding site on tubulin. Consequently, the cytotoxicity of IC261 can be attributed to the direct inhibition of microtubule polymerization rather than to the specific inhibition of CK1δ and ε [156]. These findings are supported by another study demonstrating that IC261-induced centrosome fragmentation during mitosis is independent of CK1δ [157]. Microtubule depolymerization by IC261 can furthermore be antagonized by pretreatment of cells with the stabilizing agent paclitaxel. Lower concentrations of IC261 affected dynamics of mitotic spindles resulting in cell cycle arrest and apoptosis [158]. Structural alterations of the centrosomes, centrosome amplification with the formation of multipolar spindles, and the inhibition of mitosis have already been described earlier for trophoblast cells and murine tumor cells treated with IC261 [43,46]. Interactions between IC261 and tubulin have also been characterized by a cocrystallization study confirming that the binding of IC261 roughly overlaps the colchicine binding pose and represents a new colchicine site microtubule inhibitor [159].

**Table 2:** Overview of MTAs and inhibitors of microtubule- and MAP-associated protein kinases.

Inhibitor	Target	Molecular/Therapeutic Effect	Tumor Entity	Investigation Phase	Ref.
**MSA-Taxol-domain binder**					
Paclitaxel	β-tubulin	Increase the lateral interactions between the tubulin heterodimers resulting in increased polymerization and stabilization of microtubules	Ovarian and breast cancer	Approved	[160]
Ixabepilone			Breast cancer	Approved	[126,127]
**MDA-Vinca-domain binder**					
Vincristine	β-tubulin	Lead to microtubule depolymerization by decreasing or inhibiting longitudinal interactions between tubulin heterodimers	Breast cancer, lymphomas	Approved	[161]
Vinblastine			Lymphomas, solid tumors	Approved	[161]
**MDA-Colchicine-domain binder**					
Ombra- bulin	Interface of α-/β-tubulin	Lead to microtubule depolymerization by decreasing or inhibiting longitudinal interactions between tubulin heterodimers	Ovarian cancer	Stopped in phase III	[124]
ABT-751			Lung cancer, colon cancer	Phase II	[128,129,162]
**Protein kinase inhibitors**					
Alisertib	AurA	Induce cell cycle arrest in G2/M phase, apoptosis, and autophagy; prevents AurA-induced stabilization of N-Myc	Leukemia, solid tumors	Phase III	[134,135,136,137,138,139,140,141]
AurkinA	AurA	Bind to the Tpx2-binding surface of AurA and consequently displacing AurA from the mitotic spindle	-	Preclinical	[146]
Barasertib	AurB	Decrease histone phosphorylation resulting in accumulation of aneuploidy cells and induction of apoptosis; associated with stimulation of ROS	Leukemia, solid tumors	Phase II	[142,143,144,145]
Volasertib	Plk1	Arrests cells in the G2/M phase and subsequently induces apoptosis	Leukemia	Phase III	[147,148,149]
**CK1-specific inhibitors**					
IC261	Initially designed for CK1; tubulin	Binds to tubulin resulting in direct inhibition of microtubule polymerization	Pancreatic cancer	Preclinical	[37,46,154,155,156,157,158,159]
D4476	CK1α/δ	Inhibition of CK1α/δ activity; sensitizes colorectal cancer cells to 5-fluorouracil	Colorectal cancer	Preclinical	[163]
PF-670462	CK1δ/ε	Selective inhibition of CK1δ/ε activity	Leukemia	Preclinical	[164,165]
SR-3029	CK1δ/ε	Inhibition of overexpressed CK1δ/ε	Breast cancer, skin tumor	Preclinical	[166,167]
IWP-2/IWP-4	CK1δ	Selective inhibition of CK1δ	Pancreatic, colon cancer cell lines	Preclinical	[168]
BTX-A51	CK1α/δ/ε; CDK7/9	Inhibition of CK1α and activation of p53-dependent cell death; inhibition of CDK7/9	Leukemia	Phase I	[169]
Lenalidomide	CRL4^CRBN^ E3 ubiquitin ligase; indirectly CK1α	Induces ubiquitination and degradation of CK1α	Leukemia	Approved	[170]
Umbralisib	PI3Kδ; CK1ε	Block the phosphorylation of eukaryotic translation initiation factor 4E binding protein (4E-BPI), leading to the inhibition of c-Myc translation and cell death	Lymphoma	Approved	[171,172]

### 7.3. Combination of MTAs with Additional Anticancer Agents: Advantages of Dual-Specific Inhibitors

The combination of MTAs with different anticancer agents, such as kinase inhibitors, HDACs, or DNA-damaging agents, represents an attractive antitumor strategy of the different mechanisms of action of the individual substances and the synergistic effects, which, however, often suffered setbacks in the past due to drug–drug interactions, complex application regimens, and poor patient compliance [173]. Inspired by the positive synergistic effects of multitarget strategies, dual target approaches, in particular, have been developed in recent years, largely overcoming the limitations of combination therapy and significantly reducing drug resistance and adverse effects. Dual targeting drugs are capable of interacting with two different drug targets. So far, numerous very effective synergistic dual inhibitors have been developed that interact with microtubule dynamics and with either kinases, heat shock proteins (HSPs), poly(ADP-ribose)-polymerases (PARPs), topoisomerases, HDACs, or estrogen receptors [174,175,176,177,178].

The development of highly potent dual-target drug inhibitors is most promising when functional interactions exist between the two target proteins. Thus, it is quite conceivable that the development of dual tubulin-CK1 (δ, ε, or α) inhibitors will produce significantly better synergistic effects than the use of tubulin and CK1 isoform-specific inhibitors. This is also supported by the fact that CK1 isoforms phosphorylate tubulin and MAPs are involved in vesicle transport processes along microtubules, but are also associated with the mitotic spindle apparatus, especially in cellular stress situations such as toxin exposure, mechanical damage, environmental stress exposure [44] (reviewed in [42]).

### 7.4. Modulation of the CK1 Activity with Biologicals

Apart from CK1-specific SMIs, alternative therapeutic approaches such as therapeutic peptides might be used to modulate microtubule-associated processes. By using a CK1δ-derived peptide library, a CK1δ-derived peptide encompassing amino acids 361–375 of CK1δ (P39) was identified as a prominent binding partner for α-tubulin. P39 inhibits phosphorylation of α-tubulin by CK1δ and blocks cell cycle progression of cells entering mitosis, finally leading to cell death [179]. In this context, identified peptides of a peptide library based on human CK1δ and CK1ε were used to block the interaction of CK1δ/ε with the DEAD-box RNA helicase DDX3X, which was shown to stimulate CK1 activity and Wnt/β-catenin signaling [180]. Mutations of DDX3X, which have been identified in medulloblastoma patients, increased the activity of CK1 in living cells, which led to aberrant stimulation of CK1-mediated pathways (such as Wnt/β-catenin signaling) [181]. The identified interacting CK1δ/ε-derived peptides were shown to block the activation of CK1δ/ε by DDX3X (probably caused by the inhibition of the activating interaction between both proteins) and inhibited the stimulation of CK1 activity in cell culture experiments [180].

The potential of interfering peptides was also shown by the modulation of the interaction of AXIN1-CK1ε and the regulation of CK1ε-induced phosphorylation of disheveled (DVL) and the activation of the Wnt/β-catenin signaling [182]. Furthermore, similar regulatory effects were shown for CK1α-derived peptides, which inhibit the interaction of CK1α with MDM2 leading to reduced cell viability in a p53-dependent manner [183]. Biologicals, such as these identified interaction-blocking peptides, could therefore represent promising pharmacological tools for anticancer therapy.

## 8. Conclusions

The CK1 family of serine-threonine protein kinases has a major impact on multiple cellular functions during embryogenesis and in the adult organism. Due to its role in the regulation of tubulin dynamics by phosphorylation of multiple MAPs, the deregulation of CK1 leads to human diseases, including cancer. Modulating the activity of CK1 as a promising target against tumor progression could be an interesting therapeutic approach for a multidrug treatment against tumor development. Therefore, the development of CK1 (isoform)-specific inhibitors is essential and could offer an important contribution to personalized medicine. However, the development of optimized CK1 isoform-specific compounds available for in vivo application is still challenging and should include not only the use of conventional SMIs but also dual-specific inhibitors and inhibitory peptides.

## Figures and Tables

**Table 1:** Highlighted mutations (*n* > 1) in CSNK1D and the respective cancer samples.

Mutation	Cancer
L25P	Lung Adenocarcinoma, Stomach Adenocarcinoma
G72R	Uterine Endometrioid Carcinoma, Colorectal Adenocarcinoma
E90D	Uterine Endometrioid Carcinoma, Lung Adenocarcinoma
F95I F95L	Cutaneous Melanoma Mucinous Adenocarcinoma of the Colon and Rectum (2×)
R98M R98S	Mucinous Adenocarcinoma of the Colon and Rectum Serous Ovarian Cancer
T104I T104Pfs*9	Cutaneous Squamous Cell Carcinoma, Skin Cancer, Non-Melanoma Uterine Endometrioid Carcinoma
R115H	Colon Adenocarcinoma, Head and Neck Squamous Cell Carcinoma, Uterine Endometrioid Carcinoma, Colorectal Adenocarcinoma
K122N	Endometrial Carcinoma, Lung Adenocarcinoma
R127W R127Q	Cervical Squamous Cell Carcinoma, Colorectal Adenocarcinoma Bladder Urothelial Carcinoma
R160P R160H	Colon Adenocarcinoma Colorectal Adenocarcinoma
I165T	Colorectal Adenocarcinoma, Intestinal Type Stomach Adenocarcinoma
P166H P166S	Cutaneous Melanoma Glioblastoma
R168S R168C R168H	Acute Myeloid Leukemia Skin Cancer, Non-Melanoma Uterine Endometrioid Carcinoma, Melanoma
R178W	Prostate, Colorectal Adenocarcinoma
L211F L211I	Lung Adenocarcinoma Uterine Serous Carcinoma/Uterine Papillary Serous Carcinoma
W213C W213*	Cutaneous Squamous Cell Carcinoma, Melanoma Lung Adenocarcinoma
S246=	Breast Invasive Lobular Carcinoma, Cutaneous Squamous Cell Carcinoma
E247K	Rectal Adenocarcinoma, Uterine Endometrioid Carcinoma (2×)
R256C	Angiosarcoma, Intestinal Type Stomach Adenocarcinoma
R270L R270Q	Cutaneous Melanoma (2×) Cutaneous Melanoma
R274Q	Colon Adenocarcinoma, Uterine Serous Carcinoma/Uterine Papillary Serous Carcinoma, Uterine Endometrioid Carcinoma
T344Hfs*26	Colon Adenocarcinoma, Mucinous Adenocarcinoma of the Colon and Rectum
R358Gfs*12	Colon Adenocarcinoma (3×), Uterine Endometrioid Carcinoma (2×), Cervical Squamous Cell Carcinoma
P378L	Glioblastoma, Skin Cancer, Non-Melanoma
V379Sfs*52	Breast Invasive Lobular Carcinoma
T392I	Stomach Adenocarcinoma (2×)
S411F	Cutaneous Squamous Cell Carcinoma, Bladder Urothelial Carcinoma

*—stop codon, =—splice mutation.
